# Safety Evaluation of the MiniMed 670G System in Children 7–13 Years of Age with Type 1 Diabetes

**DOI:** 10.1089/dia.2018.0264

**Published:** 2019-01-07

**Authors:** Gregory P. Forlenza, Orit Pinhas-Hamiel, David R. Liljenquist, Dorothy I. Shulman, Timothy S. Bailey, Bruce W. Bode, Michael A. Wood, Bruce A. Buckingham, Kevin B. Kaiserman, John Shin, Suiying Huang, Scott W. Lee, Francine R. Kaufman

**Affiliations:** ^1^ Barbara Davis Center for Childhood Diabetes, Aurora, Colorado.; ^2^ Edmond and Lily Safra Children's Hospital, Sheba Medical Center, Tel Aviv, Israel.; ^3^ Rocky Mountain Diabetes and Osteoporosis Center, Idaho Falls, Idaho.; ^4^ USF Diabetes Center, Morsani College of Medicine, University of South Florida, Tampa, Florida.; ^5^ AMCR Institute, Escondido, California.; ^6^ Atlanta Diabetes Associates, Atlanta, Georgia.; ^7^ University of Michigan Medical School, Ann Arbor, Michigan.; ^8^ Department of Pediatric Endocrinology, Stanford University, Stanford, California.; ^9^ SoCal Diabetes, Torrance, California.; ^10^ Medtronic, Northridge, California.

**Keywords:** Pediatric, Type 1 diabetes, Automated insulin delivery, Closed-loop insulin delivery, HbA_1c_, Glucose variability

## Abstract

***Objective:*** To evaluate the safety of in-home use of the MiniMed™ 670G system with SmartGuard™ technology in children with type 1 diabetes (T1D).

***Methods:*** Participants (*N* = 105, ages 7–13 years, mean age 10.8 ± 1.8 years) were enrolled at nine centers (eight in the United States and one in Israel) and completed a 2-week baseline run-in phase in Manual Mode followed by a 3-month study phase with Auto Mode enabled. Sensor glucose (SG), glycated hemoglobin (HbA_1c_), percentage of SG values across glucose ranges, and SG variability, during the run-in and study phases were compared. Participants underwent frequent sample testing with i-STAT^®^ venous reference measurement during a hotel period (6 days/5 nights) to evaluate the system's continuous glucose monitoring performance.

***Results:*** Auto Mode was used a median of 81% of the time. From baseline to end of study, overall SG dropped by 6.9 ± 17.2 mg/dL (*P* < 0.001), HbA_1c_ decreased from 7.9% ± 0.8% to 7.5% ± 0.6% (*P* < 0.001), percentage of time in target glucose range (70–180 mg/dL) increased from 56.2% ± 11.4% to 65.0% ± 7.7% (*P* < 0.001), and the SG coefficient of variation decreased from 39.6% ± 5.4% to 38.5% ± 3.8% (*P* = 0.009). The percentage of SG values within target glucose range was 68.2% ± 9.1% and that of i-STAT reference values was 65.6% ± 17.7%. The percentage of values within 20%/20 of the i-STAT reference was 85.2%. There were no episodes of severe hypoglycemia or diabetic ketoacidosis during the study phase.

***Conclusion:*** In-home use of MiniMed 670G system Auto Mode for 3 months by children with T1D, similar to MiniMed 670G system use by adolescents and adults with T1D, was safe and associated with reduced HbA_1c_ levels and increased time in target glucose range, compared with baseline.

## Introduction

Effective glycemic management in youth with type 1 diabetes (T1D) remains a significant challenge for families and diabetes care teams.^1–3^ Although improved intensive insulin therapies that include continuous subcutaneous insulin infusion (CSII)^4,5^ or the combination of CSII with real-time continuous glucose monitoring (CGM) (i.e., sensor-augmented pump [SAP] or sensor-integrated pump systems)^6,7^ have been shown to improve clinical outcomes in young patients with T1D, many children and adolescents have not achieved the American Diabetes Association (ADA) or International Society of Pediatric and Adolescent Diabetes (ISPAD) standards of glycemic control.^8,9^ Automated insulin delivery (AID) or closed-loop systems with algorithms that respond to real-time sensor glucose (SG) values to maintain euglycemia are, now, the forefront of technological therapies proposed to address the gaps in glycemic control.

Several short-term feasibility studies lasting from 3 days up to 8 weeks investigated early single-hormone AID systems in children and/or adolescents with T1D within clinic/hotel,^10,11^ camp,^12–14^ and in-home^15,16^ settings. These studies demonstrated improved time in target glucose range (TIR, 71–180 mg/dL), reduced time below or above target range, improved glucose variability, and/or reduced overall mean SG values compared with SAP systems or sensor-integrated pump systems with low glucose suspension. Reductions in night-time hypoglycemia with AID system use by young patients was also reported.^15,16^

The longest-duration randomized and controlled in-home study with an AID system that enrolled children as young as 6 years of age lasted 3 months and demonstrated a 24.7% increase in time spent between 70 and 145 mg/dL and a 22.9% reduction in time spent >145 mg/dL, when compared with SAP.^17^ Although mean glucose and glucose variability were significantly reduced in this study, there was no change in glycated hemoglobin (HbA_1c_) when compared with control, possibly due to closed-loop intervention only during the overnight period. More recent meta-analyses have also shown consistent improvement in 24-h day- and night-time glycemic control compared with control (i.e., SAP), and across a wide array of AID system designs and study settings.^18,19^

The MiniMed™ 670G system with SmartGuard™ technology (Medtronic, Northridge, CA) automatically adjusts basal insulin delivery based on SG values. This AID system has already been shown to safely improve HbA_1c_, TIR, and SG variability in adolescents and adults with T1D who used the system for 3 months.^20,21^ In this study, the safety and performance of in-home day-and-night use of the MiniMed 670G system for 3 months by children aged 7–13 years with T1D was evaluated.

## Methods

This nonrandomized single-arm multicenter study was conducted at nine investigational centers (eight in the United States and one in Israel) and enrolled 111 children with T1D ≥1 year, an HbA_1c_ level <10%, and a requirement of ≥8 units of insulin daily. Participants completed a 2-week baseline run-in phase in which the MiniMed 670G system (Medtronic) was in Manual Mode, followed by a 3-month study phase in which the SmartGuard Auto Mode feature was enabled. The system included the MiniMed 670G insulin pump, Guardian™ CGM system (i.e., Guardian Sensor 3 glucose sensor with Guardian Link 3 transmitter), and the CONTOUR^®^NEXT Link 2.4 blood glucose meter (Ascensia Diabetes Care, Parsippany, NJ).^20,21^

Before wearing study devices or taking part in study activities, all participants and their parent(s)/guardian(s)/companion(s) were trained on system use, as well as diabetes management principles (i.e., treatment of hyperglycemia and hypoglycemia). Participants and their parent(s)/guardian(s)/companion(s) were instructed to conduct self-monitoring of blood glucose (SMBG) measurements four to six times each day, perform system-prompted sensor calibrations, upload MiniMed 670G system data to CareLink™ Clinical software (Medtronic) every 2 weeks, and have a parent/guardian/companion ≥18 years with them during the night, for the duration of the study phase. Written informed consent and assent were obtained, in accordance with the Code of Federal Regulations (CFR) Title 21, Part 50 (United States only) and ISO14155:2011 (Europe, Middle East, and Asia only), from participants, or participants and their parent(s)/guardian(s), respectively.

Study approval was obtained from either a central or local institutional review board/ethics committee at each institution. A data safety monitoring board (i.e., data monitoring committee) was established to review data from all investigational centers and determine that all safety criteria were met across a staged enrollment during the first month of the study phase and the remainder of the study, thereafter.

Investigational staff were responsible for setting active insulin time, carbohydrate-to-insulin ratios, glucose targets, basal rates, and sensitivity factors for Manual Mode. However, Auto Mode automatically adjusted basal insulin rate based on current SG values.^22^ In Auto Mode, insulin bolus delivery was only possible through carbohydrate input or SMBG measurement for SG correction. The system's glucose target was fixed at 120 mg/dL, with an optional user-controlled temporary target of 150 mg/dL for exercise. Users could stop Auto Mode at any time, or the system could switch to Manual Mode for reasons including sensor signal loss, sensor at end of functional life, persistent glucose readings above or below prespecified limits, or an insulin delivery issue (e.g., infusion set occlusions). Participants completing the study were able to continue using the MiniMed 670G system through a voluntary Continued Access Program.

### Run-in phase

**FIG. 1.**
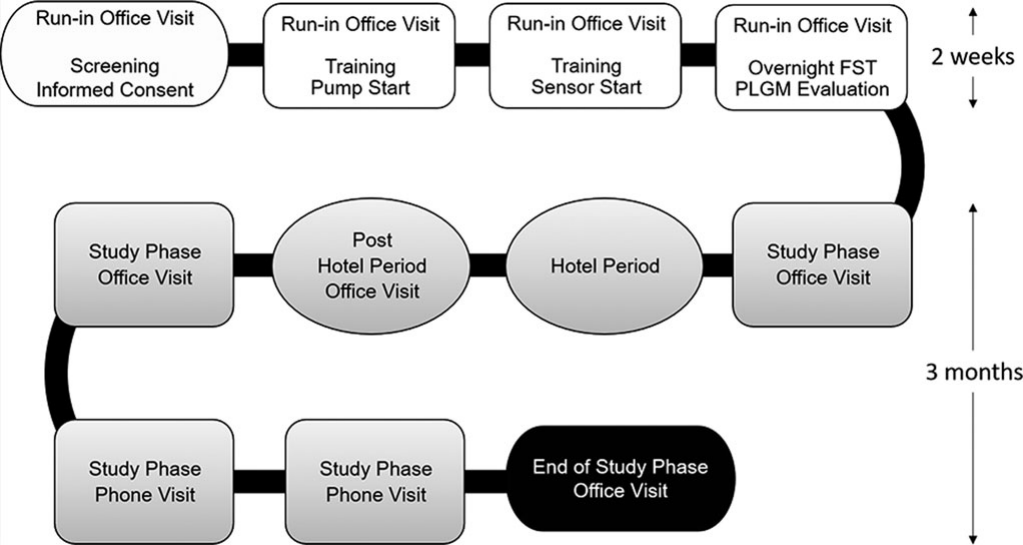
Study schedule. The run-in phase involved four visits and was 2 weeks in duration. The study phase involved six visits, including a 6-day/5-night hotel period, and was 3 months in duration. During the hotel study period, participants underwent one 24-h FST with i-STAT venous blood reference measurement. FST, frequent sample testing; PLGM, predictive low glucose management.

The baseline run-in phase lasted 2 weeks (Fig. 1) and primarily served to allow the participant(s)/parent(s)/guardian(s) opportunity to become familiar with the system. There were up to four in-clinic visits during this phase, which included screening and obtaining informed consent, study start, device training, and a final visit with overnight frequent sample testing (FST) and evaluation of the system's predictive low glucose management (PLGM) algorithm. The results of this PLGM algorithm evaluation have been reported elsewhere.^23^

During the run-in phase, the system was used as a sensor-integrated pump in the Manual Mode with the SmartGuard Suspend before low feature turned off and the Auto Mode feature not enabled. The following settings were recommended during this period: high SG alert at 300 mg/dL and low SG alert at 70 mg/dL, but not <65 mg/dL.

### Study phase

The study phase lasted 96–110 days and included three office visits, three phone visits, and one hotel period (Fig. 1). Participants used the MiniMed 670G system in Manual Mode for the first 6 days of the study phase to allow the algorithm to collect insulin utilization and CGM data to establish personalized Auto Mode initiation parameters. Participants could enable the Auto Mode feature on the seventh day after study phase entry. During Auto Mode, the recommended alert settings were the same as those for Manual Mode. The fixed alarms were set to ≤50 mg/dL, ≥300 mg/dL for 1 h, and ≥250 mg/dL for 3 h. It was recommended to have the Suspend before low feature on during Manual Mode, and for participants to use the temporary target function of 150 mg/dL during exercise.

### Hotel period

During the study phase, participants completed a hotel period that lasted 6 days and 5 nights (Fig. 1). It was conducted at a clinic, hotel, or a house, as long as investigational staffing, meal, and activity requirements were met and could be scheduled at any time during the 3-month study phase. Participants could leave to attend school but were required to return to the hotel location after school activities finished for the day. They took part in a daily exercise/activity regimen for a minimum of 4 h spread throughout the day or in the evening. The exercise/activity could include utilizing age-appropriate gym play areas and could vary at each investigational site and for each participant. Investigational staff were to be present daily for the hours of exercise/activity during the hotel period. With respect to meals, participants were allowed to eat as they normally do with meal boluses administered by the patient with staff supervision as appropriate.

Participants underwent one 24-h FST. The FST was conducted every 30 min during the night time (10:00 PM to 07:00 AM) and every 60 min during the daytime with an i-STAT^®^ system (Abbott Laboratories, Abbott Park, IL) reference or, in the event that venous access was not available, an SMBG reference. The i-STAT venous blood reference values or SMBG capillary reference values were compared with those of the Guardian Sensor 3 sensor to determine CGM system performance.

### Statistical analyses

Data regarding insulin utilization (e.g., microbolus and basal delivery) were consistently available for analysis; CGM data were occasionally unavailable due to sensor initiation warm-up periods, sensor removal, or sensor signal loss. Similar to the MiniMed 670G pivotal trial in adolescents and adults,^20,21^ exploratory analyses were conducted and *P*-values were determined without multiplicity adjustment. The primary effectiveness endpoint was changed in HbA_1c_ level, as measured by National Glycohemoglobin Standardization Program-certified central laboratories, from the baseline run-in screening/informed consent visit to the end of the 3-month study phase. The primary safety endpoints included incidence of severe hypoglycemia, diabetic ketoacidosis (DKA), serious adverse events, serious adverse device effects (SADEs), unanticipated adverse device effects (UADEs), and serious device-related adverse events.

Secondary descriptive endpoints compared between the baseline run-in and study phases included the overall mean SG; mean percentage of SG values in different glucose ranges (≤50, ≤54, ≤60, ≤70, 70–140, 70–180, >180, >250, and >300 mg/dL); overall SG variability (mean of within-day standard deviation [SD] and coefficient of variation [CV]); mean of total daily dose (TDD) of insulin; mean of basal (basal + microbolus) insulin as a percentage of TDD; and mean of body weight. For endpoints, values were averaged per subject and compared between each phase using a Wilcoxon signed-rank test or paired *t*-test. The distribution of venous i-STAT and SG values relative to the i-STAT reference values, analytical sensor accuracy (mean absolute relative difference [ARD] and 20%/20 agreement rate), mean number of hyperglycemic events (i.e., two or more consecutive SG values greater than a specified threshold of >180, >250, and >300 mg/dL), and mean number of hypoglycemic events (i.e., two or more consecutive SG values ≤50, ≤54, ≤60, and ≤70 mg/dL) during the hotel FST was also determined. All analyses were based on the 105 participants completing the study phase. Statistical analyses were performed using SAS^®^ 9.4 (SAS Institute, Cary, NC).

## Results

### Participants and system use

**FIG. 2.**
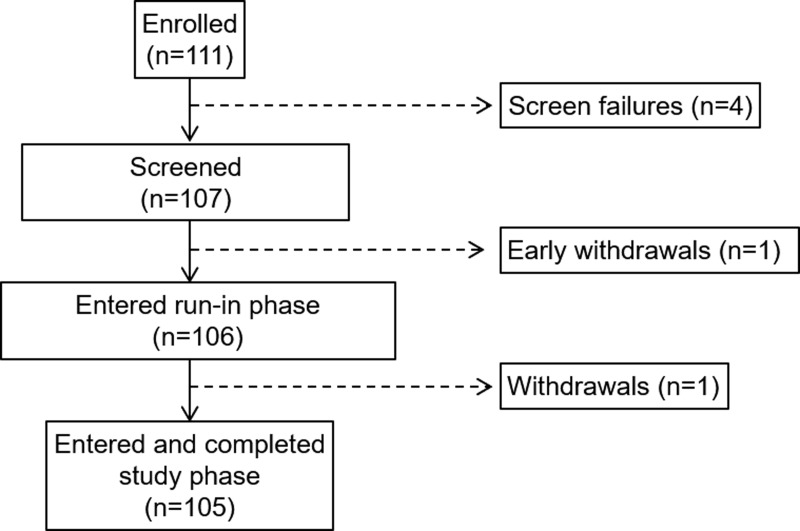
Disposition of study participants.

Of the 111 subjects enrolled and ranging from 7 to 13 years of age, there were four screen failures, one withdrawal before the run-in phase, and one withdrawal during the run-in phase (Fig. 2). There were no withdrawals during the study phase. The final number of participants completing the study phase, as well as the hotel period with FST, was 105 (mean ± SD age of 10.8 ± 1.8 years) and their baseline characteristics are listed in Table 1. Of all the participants completing the study phase, 102 entered the voluntary Continued Access Program.

**Table 1.** Study Participant Characteristics at Baseline

	*Participants* N* = 105*
Age, years	10.8 ± 1.8 (11.0, 7–13)
Female, *n* (%)	49 (46.7)
Male, *n* (%)	56 (53.3)
Weight, kg^*^	42.8 ± 13.0 (40.1, 23.4–83.0)
BMI, kg/m^2^^*^	19.1 ± 4.3 (18.0, 14.0–41.0)
BMI *z*-score^*^	0.3 ± 1.0 (0.2, −2.0–2.7)
TDD, U/(kg·d)^*^	0.8 ± 0.2 (0.8, 0.4–1.3)
Duration of diabetes, years	5.6 ± 2.9 (4.9, 1.1–13.0)

All values are shown as mean ± SD (median, min–max) excluding gender.

^*^ One participant's height and weight were not measured at enrollment.

BMI, body mass index; SD, standard deviation; TDD, total daily dose of insulin.

### Glycemic control, insulin delivered, and body weight

The Auto Mode feature was used a median of 80.6% (interquartile range [IQR], 70.0%–87.7%) of the time or 19.3 h/day (IQR, 16.8–21.1 h/day). The sensor was used a median of 90.9% (IQR, 86.2%–93.9%) of the time or 21.8 h/day (IQR, 20.7–22.5 h/day). The mean ± SD HbA_1c_ dropped from 7.9% ± 0.8% at baseline run-in to 7.5% ± 0.6% by the end of the study phase (Table 2, *P* < 0.001). Stratification of participants by HbA_1c_ level during the run-in and study phases revealed that >50% of study participants had an HbA_1c_ level ≤7.5% by the end of the study compared with only 36% at baseline run-in. Overall mean SG reduced from 169 ± 22 to 162 ± 12 mg/dL (*P* < 0.001^†^) and the mean percentage of SG values below, within, and above target glucose ranges were all improved, compared with baseline (Table 2). The increased TDD observed from baseline to the end of study appeared to be due to an increase in both basal and bolus insulin delivery.

**Table 2.** Glycated Hemoglobin, Sensor Glucose, Glycemic Control, Variability, Insulin Delivered, and Body Weight During the Run-In and Study Phase

	*Run-in phase*	*Study phase*	P
HbA_1c_, %	7.9 ± 0.8 (7.9, 7.2–8.4)	7.5 ± 0.6 (7.5, 7.1–7.8)	<0.001
SG, mg/dL	169 ± 22 (168, 155–184)	162 ± 12 (162, 154–169)	<0.001^†^
Percentage of sensor glucose values across ranges, mg/dL			
≤50	0.8 ± 1.2 (0.5, 0.1–1.1)	0.5 ± 0.5 (0.4, 0.2–0.8)	0.0012^†^
≤54	1.3 ± 1.5 (0.8, 0.3–2.0)	0.8 ± 0.7 (0.7, 0.3–1.1)	<0.001^†^
≤60	2.2 ± 2.3 (1.4, 0.7–3.3)	1.4 ± 1.0 (1.2, 0.7–1.9)	<0.001^†^
≤70	4.7 ± 3.8 (3.5, 1.8–7.2)	3.0 ± 1.6 (2.9, 1.8–3.8)	<0.001^†^
>70–140	35.0 ± 10.5 (33.5, 27.8–43.7)	42.6 ± 6.3 (42.5, 38.6–46.0)	<0.001
>70–180	56.2 ± 11.4 (55.9, 50.2–63.4)	65.0 ± 7.7 (64.6, 60.3–70.4)	<0.001
>180	39.1 ± 12.8 (38.4, 31.4–47.7)	32.0 ± 7.7 (32.4, 26.5–36.8)	<0.001
>250	13.3 ± 7.7 (11.5, 7.5–18.3)	10.3 ± 5.1 (9.8, 6.4–12.9)	<0.001^†^
>300	4.7 ± 3.8 (3.7, 1.8–7.0)	3.7 ± 2.7 (3.1, 1.7–4.9)	0.0037^†^
Within-day SD of SG, mg/dL	57.7 ± 8.3 (58.4, 52.2–65.0)	54.7 ± 7.5 (55.0, 49.7–59.1)	<0.001
Within-day CV of SG, %	34.8 ± 4.3 (34.4, 32.0–37.8)	33.7 ± 3.1 (33.7, 31.4–35.8)	0.0023
TDD, U/(kg·d)^*^	0.8 ± 0.2 (0.8, 0.7–0.9)	0.9 ± 0.2 (0.8, 0.7–0.9)	0.0037^†^
Basal insulin as % of TDD^*^	44.5 ± 7.4 (45.3, 39.1–49.9)	44.0 ± 7.3 (44.7, 40.3–47.8)	0.7281^†^
Weight, kg^*^	42.8 ± 13.0 (40.1, 32.4–51.6)	44.9 ± 13.4 (42.3, 33.8–53.3)	<0.001^†^

The run-in phase duration was 2 weeks and the study phase duration was 3 months. All values are shown as mean ± SD (median, interquartile range).

^*^ One participant's height and weight were not captured at enrollment.

^†^ Wilcoxon signed-rank test.

CV, coefficient of variation; HbA_1c_, glycated hemoglobin; SG, sensor glucose; TDD, total daily dose of insulin, includes basal + microbolus.

**FIG. 3.**
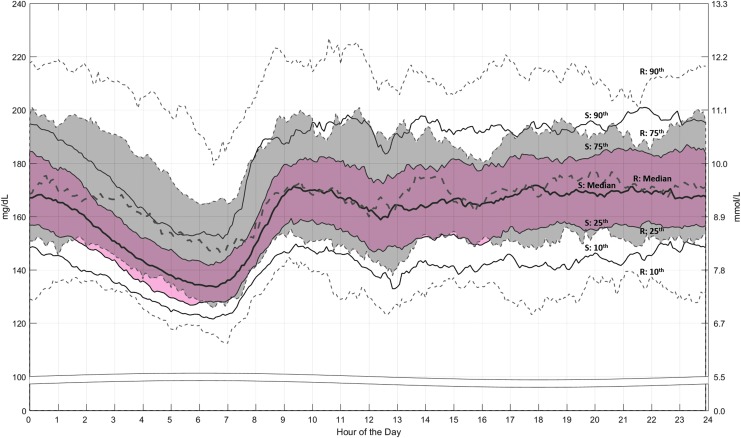
Sensor glucose profile during the run-in and study phases. The median and 10th, 25th, 75th, and 90th percentile ranges of sensor glucose values (mg/dL on the left axis and mmol/L on the right axis) throughout the 24-h period are shown for the run-in (R) phase (gray band and dashed lines) and study (S) phase (pink band and solid lines).

The 24-h profile of the median and percentile ranges of SG values (Fig. 3) shows that SG was predominantly within the hyperglycemic range during the early morning to midnight periods of baseline run-in. However, both were reduced during the Auto Mode-enabled study phase. The within-day SD and CV of SG values decreased from 57.7 ± 8.3 to 54.7 ± 7.5 mg/dL (*P* < 0.001) and 34.8% ± 4.3% to 33.7% ± 3.1% (*P* = 0.0024), respectively, supporting the study phase 24-h profile. Study phase reductions in the mean percentage of SG values below and above target glucose range, increases in TIR, and decreases in SG variability were also observed during the night time (10:00 PM to 07:00 AM) period (Supplementary Table S1; Supplementary Data are available at https://www.liebertpub.com/suppl/doi/10.1089/dia.2018.0264).

The effect of the system on fasting glucose levels was also evident, where the mean overall fasting SMBG between 05:00AM and 09:00AM decreased from 162 mg/dL at baseline to 158 mg/dL (*P* = 0.23) during the Auto Mode-enabled study phase. The percentage of study phase fasting SMBG values <70 mg/dL was reduced (from 3.2% ± 9.0% to 1.2% ± 2.5%, *P* = 0.126; Wilcoxon signed-rank test.), that within 70–180 mg/dL was increased (from 59.7% ± 26.7% to 71.9% ± 16.3%, *P* < 0.001; Wilcoxon signed-rank test.), and that >180 mg/dL was reduced (from 37.1% ± 27.1% to 26.9% ± 16.3%, *P* < 0.001; Wilcoxon signed-rank test.).

### Hotel period FST with i-STAT reference

During the hotel period, the percentage of within-target SG values was 68.2% ± 9.1% and that of the i-STAT reference values was 65.6% ± 17.7% (Table 3). For the hotel FST, the overall mean ± SD number of events within hypoglycemic ranges (≤50, ≤54, ≤60, and ≤70 mg/dL) for the 105 FST days was ≤0.1 ± 0.3. The overall number of events >180 mg/dL was 1.4 ± 0.6; >250 mg/dL was 0.6 ± 0.5; and >300 mg/dL was 0.2 ± 0.4. In addition, the overall CGM system accuracy, as demonstrated by the mean ARD between SG and i-STAT reference values, was 11.9% ± 13.5% (3271 paired points) and the percentage of SG values within 20%/20 of i-STAT reference values was 85.2%.

**Table 3.** Distribution of i-STAT and Sensor Glucose Values During the Hotel Period Frequent Sample Testing

	*i-STAT*		*Sensor*	
*Reference glucose*	N	*%*	N	*%*
≤70 mg/dL	34	1.1 ± 2.1 (0.0, 0.0–2.9)	4362	2.5 ± 2.0 (1.9, 1.1–3.3)
>70–180 mg/dL	2224	65.6 ± 17.7 (68.8, 52.9–78.8)	118,395	68.2 ± 9.1 (67.8, 62.9–75.3)
>180 mg/dL	1104	33.3 ± 17.9 (29.4, 20.6–46.9)	50,711	29.3 ± 9.4 (29.2, 22.4–34.8)

The mean ± SD (median, min–max) percentage of glucose values are shown.

N, number of i-STAT reference and sensor values.

### Safety

MiniMed 670G system use from baseline run-in to study end comprised a total of 13,738 patient days. There were no episodes of severe hypoglycemia, no SADEs, UADEs, or serious device-related adverse events during the study. During the run-in phase, there were 27 severe hyperglycemia episodes (0.89 per 100 patient days); during the study phase, there were 76 (0.71 per 100 patient days). There was one episode of DKA that occurred before MiniMed 670G system use. This patient was hospitalized, discharged after 1 day, and withdrawn from the study per protocol.

## Discussion

This study, with >13,000 patient days of in-home MiniMed 670G system use by children with T1D, showed that the system is safe to use and that there were no severe hypoglycemic events or episodes of DKA during the 3-month study phase. Similar to in-home system use by adolescents and adults with T1D,^20,21^ there were no serious device-related adverse events. Auto Mode was used an overall median of 80.6% of the time by children, compared with the medians of 75.8% and 88.0% for adolescents and adults, respectively.^21^ The slightly higher usage in children versus adolescents (80.6% vs. 75.8%) is most likely attributed to the protocol-required parental/guardian involvement during the study, and to normal parental supervision of this young age group. In this study, the average number of Auto Mode exits per patient was 5.8 ± 1.6 per week; the majority of which were due to prolonged high glucose, sensor integrity check failure, or missed calibration. Greater sensor usage was also observed for children, where sensors were used a median of 90.9% (21.8 h/day) of the time. In adolescents it was 88.6% (21.3 h/day), and in adults it was 93.1% (22.3 h/day).^21^ In addition to the established safety of the system, improved glycemic management was observed, with children having an increased TIR and less time spent in both hypoglycemia (−1.7%) and hyperglycemia (−7.1%) when in Auto Mode versus open-loop Manual Mode.

Reduced HbA_1c_ was also observed in this well-controlled group, as demonstrated by baseline run-in HbA_1c_ levels within the HbA_1c_ goals established by the ADA and ISPAD for children in a similar age range.^8,9^ In-home use of the MiniMed 670G system for 3 months appeared to show baseline HbA_1c_ level-dependent improvements in HbA_1c_ whereby levels <7.0% were increased by 0.4%; levels 7.0%–8.0% were reduced by 0.2%; and levels >8.0% were reduced by 0.7%. It remains to be investigated if greater improvement could be observed in young patients with higher HbA_1c_ levels at baseline.

The day-to-day and within-day variability of glucose levels in youth with T1D can be an ongoing issue for diabetes care teams. This is important, as achieving HbA_1c_ goals in youth with T1D,^3,24^ while mitigating the incidence and risk of hypoglycemia,^25^ can be challenging, if not burdensome, for the multiple individuals involved in managing the daily well-being of young patients with T1D.^26–28^

Effectively maintaining euglycemia during critical developmental stages, and into puberty and adulthood, are key to reducing a series of diabetes-related complications.^29,30^ Glycemic variability involving long-term hyperglycemia can increase cardiovascular risk and the incidence of retinopathy and renal failure. Increased exposure to hypoglycemia, which can be difficult to ascertain in children, is reported to be associated with long-term cognitive deficits^31–33^ and seizure.^34,35^ In this study, glucose variability, especially within the hyperglycemic ranges, was lowered throughout the 24-h day during the Auto Mode-enabled study phase when compared with baseline. This was demonstrated by reduced within-day SD and CV of SG levels. The reduction in median SG levels was most pronounced during the morning hours beginning as early as midnight, and was supported by lowered fasting SMBG values. The overall reduction in glucose variability was associated with an increase in TDD, with primarily no change in the percentage of basal insulin delivery. Although increased TDD and reduced glucose variability have been previously reported for adolescents during AID system use,^21^ basal insulin delivery has been shown to either decrease^21^ or increase^16^ compared with baseline or control. All together, this appears to demonstrate AID systems effectively addressing glycemic need.

Similar to previously reported AID system use for at least 3 months in adolescents or adults,^21,36^ this study shows that 3-month MiniMed 670G system use in children increased time in target glucose range and reduced SG variability and HbA_1c_ levels compared with 2-week open-loop therapy. Nevertheless, established safety and utility of AID system use have been equally important.^37,38^ A small short-term (3 days) randomized controlled trial (RCT) in children (aged 5–8 years) demonstrated improved TIR, lowered SG levels, and a comparable rate of hypoglycemia versus control with an AID system previously used in older cohorts, but modified with password-protected lockout screens that changed daily.^39^

A small longer-duration (21 days) in-home RCT in slightly older youth (10–18 years) investigated AID in a system with contingencies similar to some of those inherent to the algorithm of this study's system (i.e., maximum insulin infusion limit, insulin delivery suspension during rapid rates of decreasing SG, and automated reestablishment of open-loop settings in situations of sensor signal loss).^16^ The aforementioned investigational AID system increased TIR, reduced time spent above target, and lowered mean SG levels, whereas time spent in hypoglycemia remained comparable, versus the SAP control. Similarly, increased TDD compared with control, mainly due to increased insulin delivery and concomitant variability in insulin delivery, was also observed.^16^

These insulin delivery findings with closed-loop system use are not unusual in young patients with T1D who may not be receiving sufficient insulin to reach appropriate glycemic control; as indicated by higher baseline hyperglycemia, glucose variability, and HbA_1c_ levels. Use of the same system in adolescent patients increased TIR and reduced the time spent above target, compared with SAP control, without changing glucose variability or TDD.^40^ Although the AID system was used for only 7 days, the lack of change in TDD was described as being partly due to the offsetting of total daily basal and bolus insulin delivered.

The percentage of SG values within target glucose range during the study phase for the pediatric cohort (65.0% ± 7.7%) was lower when compared with that for adolescents (67.2% ± 8.2%), as well as adults (73.8% ± 8.4%), using the same system.^21^ This may be, in part, due to differences in levels of development, catabolism, types and quantity of food consumed, and physical activity. Although the overall mean ARD of 11.9 ± 13.5 (3271 paired points) for the system in the pediatric cohort was higher than the 11.2% ± 9.7% (902 paired points) reported for adolescents^21^ and the 10.0% ± 8.7% (2808 paired points) reported for adults,^21^ overall clinical accuracy relative to the gold standard Yellow Springs Instruments reference demonstrated in the Guardian CGM system's pivotal trial was 10.9% ± 10.7%.^41^

This study was a single-arm nonrandomized design with a 2-week baseline run-in duration, similar to the 3-month investigation of the MiniMed 670G system in adolescents and adults.^21^ Limitations to the study design that preclude generalizations of findings include the absence of a control group, different time durations for baseline run-in and study phase, and the exclusion of patients with HbA_1c_ levels >10.0% and a recent history of DKA, or two or more episodes of severe hypoglycemia resulting in coma or seizure. The concomitant involvement of parents/guardians, which is a standard practice for pediatric studies, may have resulted in greater than typical influence for a clinical trial involving a novel medical device. Nevertheless, the AID system in this study is currently CE-marked and approved in the United States for patients with T1D aged ≥7 years.

The pivotal trial of the MiniMed 670G system in adolescents and adults demonstrated improvement in multiple outcomes of glycemia, an important finding also observed in this study of >100 pediatric patients with T1D who had a higher mean HbA_1c_ level at baseline. An analysis to determine the effect of participant gender or ethnicity on outcomes was not planned within the study design. Approximately 97% of pediatric participants completing this study continued to use the MiniMed 670G system in the voluntary Continued Access Program, ∼15% more than that observed for adolescents and adults completing the first pivotal trial, indicating high satisfaction with this AID system.

Although the MiniMed 670G system pivotal trial in the older age groups^21^ and this study in pediatric participants reported no events of DKA and no episodes of severe hypoglycemia or serious device-related adverse effects during the Auto Mode-enabled study phase, a randomized investigation of MiniMed 670G system use versus SAP, MDI, and CSII therapies is planned to evaluate long-term safety and efficacy of the system (NCT02748018). To date, this study is the longest in-home day and night AID study in children as young as 7 years. Safety of the system in a subgroup of children as young as 2 years is ongoing.

Findings reported here suggest that in-home use of the MiniMed 670G system with the SmartGuard Auto Mode feature by children with T1D, similar to that observed for adolescents and adults with T1D, safely improves overall glycemic control by reducing glucose variability and minimizing hypo- and hyperglycemia.

## Supplementary Material

Supplemental data
