# Supplementary material for: Safety Evaluation of the MiniMed 670G System in Children 7–13 Years of Age with Type 1 Diabetes
Source: Diabetes Technol Ther. 2019 Jan 7;21(1):11–9. doi: 10.1089/dia.2018.0264 (PMC6350071; doi:10.1089/dia.2018.0264)
Supplement: Supplemental data [file Supp_Table1.pdf]

Supplementary Data

Supplementary Table S1. Night-Time Sensor Glucose, Glycemic Control, Variability, and Insulin Delivered During the Run-In and Study Phase

	Run-in phase	*Study phase*	P
SG, mg/dL	166 ± 26 (163, 148–184)	155 ± 13 (153, 145–162)	<0.001
Percentage of sensor glucose values across ranges, mg/dL			
≤50	1.0 ± 1.6 (0.3, 0.0–1.2)	0.5 ± 0.6 (0.3, 0.1–0.7)	0.0125[†]
≤54	1.5 ± 2.2 (0.5, 0.0–2.0)	0.7 ± 0.7 (0.5, 0.2–1.0)	<0.001[†]
≤60	2.4 ± 3.2 (1.2, 0.4–3.5)	1.2 ± 1.0 (0.8, 0.4–1.6)	<0.001[†]
≤70	4.9 ± 5.2 (3.3, 1.4–7.4)	2.4 ± 1.6 (1.9, 1.1–3.5)	<0.001[†]
70–140	35.4 ± 14.7 (35.0, 24.6–46.2)	48.6 ± 8.1 (48.9, 42.4–54.1)	<0.001
>70–180	56.6 ± 15.3 (57.5, 47.7–67.4)	70.9 ± 9.4 (72.0, 64.3–77.6)	<0.001[†]
>180	38.5 ± 16.7 (37.6, 27.8–49.0)	26.7 ± 9.0 (25.7, 20.1–33.1)	<0.001
>250	11.7 ± 8.9 (9.7, 4.7–17.4)	7.8 ± 5.1 (6.8, 3.6–11.0)	<0.001[†]
>300	3.8 ± 4.0 (2.6, 0.4–5.7)	2.6 ± 2.4 (1.9, 0.8–3.5)	<0.001[†]
Within-day SD of SG, mg/dL	42.1 ± 7.9 (43.2, 37.0–47.4)	39.6 ± 6.3 (39.1, 34.8–44.2)	<0.001[†]
Within-day CV of SG, %	26.2 ± 4.7 (25.6, 23.2–29.5)	25.5 ± 3.0 (25.6, 23.6–27.4)	0.1079
TDD, U/(kg·d)*	0.2 ± 0.1 (0.2, 0.2–0.2)	0.2 ± 0.0 (0.2, 0.2–0.2)	0.0572
Basal insulin as % of TDD*	72.2 ± 11.7 (73.8, 63.9–82.7)	75.9 ± 7.9 (76.5, 72.5–82.0)	<0.001

The run-in phase duration was 2 weeks and the study phase duration was 3 months. All values are shown as mean ± SD (median, interquartile range).

*One participant's height and weight were not captured at enrollment.

[†]Wilcoxon signed-rank test.

CV, coefficient of variation; SD, standard deviation; SG, sensor glucose; TDD, total daily dose of insulin, includes basal + microbolus.
